# New Way to Apply Cocain

**Published:** 1907-03-15

**Authors:** F. Touchard

**Affiliations:** Professor in the Ecole Dentaire of Paris


### NEW WAY TO APPLY COCAIN.

BY F. TOUCHARD,

Professor in the Ecole Dentaire of Paris.

In a communication to the Academy of Medicine the author says that recent discussions in that society as to the value of cocain do not seem to have modified the opinions either of its admirers or of its detractors. He himself has for some time been an advocate of the employment of cocain anesthesia by the dentists, and anticipates that the drug will be of still more value to the profession.

Up to the present, so far as known, the analgesic action of cocain has only been applied in the extraction of teeth, the principal indications for its use being timidity of the patient or difficulty of extraction. Cocain remains, the author claims, the favorite analgesic, as it can be used always and everywhere—its administration, when regulated by a detailed but non-complicated technique, insuring the elimination of failure or accident.

The pain of extraction will be of short duration, if there be no complications; but if the conformation of the tooth require separation of the roots, or prior operative procedures render the extraction more laborious, it is otherwise, and then the value of anesthesia will be appreciated. The duration of cocain anesthesia is here an important factor. The general anesthetics employed in dental practice—such as nitrous oxide and somnoform—can be administered only for a limited period of anesthesia—too short for our purpose. Again, the administration of chloroform or ether offers inconveniences in dental practice that offset any advantages that might be obtained.

In the presence of convincing results in one thousand cases of extraction, it seems to the author but natural that the benefits of cocaine anesthesia should be utilized in other cases.

M. Reclus, in 1894, in his book on the use of cocain in surgery, stated the problem clearly and was astonished that the very simple procedure was not taken advantage of in current practice. He says: "Dentists, in having recourse to cocain, anesthetise the tissues when they desire to extract a tooth, yet they abstain from its use in those painful cases in which they either extract or destroy the pulp by means of broaches or caustics. Why not, then, practise deep injection to the level of the roots? Certainly these injections prove efficient. They so act upon the nerves that lead to the pulp that although in extraction these nerves become stretched and torn, the patient does not experience the slightest suffering".

The author does not think that the advice of M. Reclus has been taken advantage of and applied with definite purpose for the alleviation of pain as it might have been in the treatment of various pathological conditions. We have hesitatingly made the experiment of bringing a relatively concentrated solution of cocain into direct contact with a diseased pulp. The same is true as to the addition of cocain to other preparations which have a rather variable action with the idea of suppressing the pain that makes their use necessary—as in devitalisation of the pulp. The application of tampons of cotton soaked in a 10 percent cocain solution to modify the sensitivity of painful dentine in caries of the second degree has but a doubtful influence even after twenty minutes' contact.

The indications and counter-indications for the use of cocain seem clearly established by the following case: A patient called to have a tooth extracted. Upon examination it was found that the tooth required simple conservative treatment, the caries being only of the second degree. The patient was nervous and timid, and the pain in the tooth was so acute that she insisted upon its extraction, because of her fear that the pain should increase. Injections of cocain were made in the gum as is usual in the technique of extraction. Now, while the cocain was acting, the patient became comfortable, and decided to retain her tooth. Accordingly, beginning the work of excavation for a filling operation at once, the operator was much surprised as was also the patient—to find that the contact of the instruments, which had previously caused so much pain, was felt merely as a vague sensation of touch. The diseased dentine was removed, and the cavity at once prepared and then filled, all being effected without causing the slightest pain.

Greatly impressed thereby, Professor Touchard resolved to apply cocain anesthesia to any of the various painful conditions he might be called upon to deal with. The treatment was experimentally applied in the most diverse cases, with excellent results; he now feels that he possesses a simple and practical method of assuaging pain, by means of which the needed treatment of dental caries may be rapidly accomplished. In hypersensitiveness either of the teeth or pulp, particularly in the case of nervous patients, it is an advantage to have recourse to cocain anesthesia.

He tabulates twenty observations, taken from fifty cases and says: "The reading of the annexed observations will make this latter point clearer: You can follow the progress of insensitivity step by step, and although too much stress may seem to be laid on this point, it is a fact that no matter what the work to be done or condition present—excavation of softened and sensitive dentine, removal of diseased pulp whether inflamed or not, extraction of the contents of root-canals—no matter which tooth was treated, satisfactory results were obtained."

The degree of emotion in the patient, particularly in women was an additional reason for resorting to analgesic methods, and he has not found that such a course offers the slightest inconvenience, the patient becoming calm from the moment of realising cessation of pain. He has not only not observed any accidents, but even any untoward incidents whatever due to the action of cocain.

He feels that these uniformly successful results are due to the application of a well-regulated technique, and gives the following as his own procedure: The solution employed is a weak one, as advised by M. Reclus. A mixture of cocain hydrochloride and eucaine is generally used, as follows: 1 cc. of the solution contains 15 mgm. of cocain hydrochloride and 5 mgm. of beta-eucaine, the latter being added to counteract the vaso-constrictor action of the cocain. Recourse has likewise been successfully had to the oily solution of pure cocain, as suggested by Poinsot.

Finally ,in the case of a highly congested condition of the diseased parts, rendering the effect of the cocain on the inflamed areas uncertain, and the best results have been secured from the addition of adrenaline to the cocain. Adrenaline seems to limit, and thereby reinforce the action of the cocain; its action may be likened to that of the elastic ligature placed at the base of a finger into which cocain is to be injected. Adrenaline serves as a vascular ligature, so to speak.

Two conditions must be observed in order to obtain complete anesthesia: By means of injections circumscribe carefully the tooth to be extracted, and ascertain accurately just when complete anesthesia is established. The majority of the failures registered against cocaian are due, he believes, to the non-observanse of these details.

The technique of the injections is likewise important attention should be given to every minute detail. It is not always an easy matter to make an injection in just the right place, in tissues which are frequently no thicker than the diameter of the needle. Steel needles should be used, very fine and sharp pointed. Needles of platino-iridium, although their sterilisation by means of the flame is very easy, have the inconvenience of being less keenly pointed, and they appear, when seen in the same lighting, of heavier diameter than the steel needles. Steel needles should be sterilized by immersion in chloroform for a quarter of an hour—or, better, in a drying stove.

The injection becomes more difficult in proportion to the depth of the root; it is also more difficult when upon the external surface than upon the internal, and in the lower than in the upper jaw. It should be made slowly, the needle being inserted more and more deeply until it has penetrated to a depth of about 2 cm. in the direction of the apex of the root, keeping it as near the periosteum as possible. If the injection has been well made it will not be long before the gum assumes a characteristic whitish tint, and at the same time its sensibility disappears. The sensibility of the tooth subsides gradually through a period of time varying from two to fifteen minutes. The anesthesia lasts from ten to forty minutes—sometimes more—gradually disappearing, the first part to come from under its influence being the most sensitive, viz., the pulp. While it lasts the operator usually has time for the performance of the otherwise painful parts of the operation; but if further anesthesia be necessary another injection will supply it providing that the prescribed danger zone be not overstepped *i.e.*,that the total amount of cocain injected does not exceed the safe physiological dose. A cocain injection not exceeding a total dose of 0.05 gramme in a single sitting will occasion neither accidents nor incidents; still less will there be reason for fear if a weaker solution be used. The after-sensations following the operation are only such as would follow any operation, whether cocain be used or not. The injuries inflicted on live tissues by the procedures necessary in an ordinary filling are always followed by a few hours of discomfort, rather than pain. What is of importance to know is that the rapidity of cocain treatment—which to some extent enables us to surmount obstacles—by no means implies any increase of subsequent pain.

The exact physiology of dental anesthesia by cocain is still to be established. Taking into consideration the vascular and nervous wealth of the periosteum, its connection with the gum on the one side and with Tomes' fibrils on the other, and the latter in their turn with the pulp there is every reason to believe that cocain acts gradually, anesthetising first the peripheral parts and reaching the centre last. In favor of this hypothesis is the fact that after a gingival injection the dentine is anesthetised before the pulp. As regards the exact action of cocain on the nerve elements, as indicated by M. Dastre, it is comparable to a "curare" acting upon the nerve terminals. The ignorance under which we labor as to where the nerve fibrils of the tooth end forces us to remain without complete demonstration of this. Possibly cocain may act directly on all the dental tissues by suspending the activity of the cell in each case. Even though the exact progress of the anesthesia be not scientifically established, the anesthesia itself is practically apparent in the whole extent of the tooth.

In two cases of failure reported, both being cases of acute pulpitis, the cocain injection anesthetised the dentine, and the pulp was reached without pain; but as the latter remained sensitive, recourse was had to slow devitalisation by means of arsenious acid. In these two cases the solution of cocain and eucaine was used. In two other cases of acute pulpitis, however, afterwards treated, complete anesthesia of the pulp was obtained with a mixture of adrenalin and cocain.

The author considers that counter-indications against the employment of this method—the use of which he proposes to make general—are but few. They are such as apply to cocain in general, and are of course lessened when weak doses are employed.

In cases of gingivo-periostitis, cocain injection will render but little service; this matters little, however, as treatment of the tooth in such cases is seldom painful. Adrenalin, modifying the circulation of the inflamed tissues, would assist the action of cocain; hence the good results obtained in cases of acute pulpitis. On the other hand, it would be useless to have recourse to cocain anesthesia in the treatment of caries of the fourth degree. The tooth being dead its treatment would be painless even without an anesthetic.

He sums up the advantages to be obtained from gingival cocain injections in painful dental operations as follows:

### Conclusions.

(1) It always suppresses pain, often as intense as that produced by the extraction of a tooth, and certainly for a longer period than is required for an extraction.

(2) In caries of the second degree the pain otherwise experienced in the preparation is avoided. The means ordinarily applied to obtain insensibility of the dentine have but little efficacy. The treatment under cocain anesthesia is rendered more rapid, as it requires but one sitting to accomplish what could otherwise have been done—and that less perfectly—only in several treatments. Cocain anesthesia permits the painless preparation of rentention points in the case of a gold filling.

(3) In caries of the third degree, the pulp being anesthetised, one can at once proceed with the treatment. Cauterization is avoided, and the operation is rendered briefer.

The observations tabulated by the author apply to cases of caries of the second and third degrees. Two were deemed to be failures—only relative failures, as the dentine was anesthetised. Attention is called to a case in which an injection of cocain adrenalin was successful when employed one week after cocain by itself had failed.

The duration of anesthesia was not always recorded in these cases, but it was practically maintained for a longer period than would have been necessary for the performance of the cavity preparation.

M. Touchard has frequently had to treat females of highly nervous temperament where the lesions were very painful; and the results obtained in such cases were all the more satisfactory.—*L'Odontologie.*

